# The Clinical Impact of Methotrexate-Induced Stroke-Like Neurotoxicity in Paediatric Departments: An Italian Multi-Centre Case-Series

**DOI:** 10.3389/fneur.2022.920214

**Published:** 2022-06-10

**Authors:** Andrea Santangelo, Emanuele Bartolini, Giulia Nuzzi, Thomas Foiadelli, Alexandre Michev, Tommaso Mina, Irene Trambusti, Valeria Fichera, Alice Bonuccelli, Gabriele Massimetti, Diego G. Peroni, Emanuela De Marco, Luca Coccoli, Laura Luti, Sayla Bernasconi, Margherita Nardi, Maria Cristina Menconi, Gabriella Casazza, Dario Pruna, Rosamaria Mura, Chiara Marra, Daniele Zama, Pasquale Striano, Duccio M. Cordelli, Roberta Battini, Alessandro Orsini

**Affiliations:** ^1^Paediatric Neurology, Paediatric Department, Santa Chiara University Hospital, Azienda Ospedaliero Universitaria Pisana, Pisa, Italy; ^2^Department of Developmental Neuroscience, Istituto di Ricerca e Cura a Carattere Scientifico (IRCCS) Fondazione Stella Maris, Pisa, Italy; ^3^Clinica Pediatrica, Fondazione IRCCS Policlinico San Matteo, Pavia, Italy; ^4^Paediatric Haematology/Oncology Department, Fondazione IRCCS Policlinico San Matteo, Pavia, Italy; ^5^Department of Clinical and Experimental Medicine, University of Pisa, Pisa, Italy; ^6^Paediatric Oncology and Haematology Department, Santa Chiara Hospital, Azienda Ospedaliero Universitaria Pisana, Pisa, Italy; ^7^Paediatric Neurology, Paediatric Department, ARNAS G. Brotzu, Cagliari, Italy; ^8^Paediatric Oncology and Haematology, Pediatric Department, ARNAS G. Brotzu, Cagliari, Italy; ^9^Paediatric Unit, IRCCS Azienda Ospedaliero-Universitaria di Bologna, Bologna, Italy; ^10^Paediatric Neurology and Muscular Diseases Unit, IRCCS Istituto Giannina Gaslini, Genova, Italy; ^11^Department of Neurosciences, Rehabilitation, Ophthalmology, Genetics, Maternal and Child Health, University of Genova, Genova, Italy; ^12^Unitá Operativa Complessa (UOC) Neuropsichiatria dell'età Pediatrica, IRCCS Istituto delle Scienze Neurologiche di Bologna, Bologna, Italy

**Keywords:** stroke-like syndrome, methotrexate, pseudo-stroke, neurotoxicity, subacute toxicity

## Abstract

**Introduction:**

Stroke-like syndrome (SLS) is a rare subacute neurological complication of intrathecal or high-dose (≥500 mg) Methotrexate (MTX) administration. Its clinical features, evoking acute cerebral ischaemia with fluctuating course symptoms and a possible spontaneous resolution, have elicited interest among the scientific community. However, many issues are still open on the underlying pathogenesis, clinical, and therapeutic management and long-term outcome.

**Materials and Methods:**

We retrospectively analyzed clinical, radiological and laboratory records of all patients diagnosed with SLS between 2011 and 2021 at 4 National referral centers for Pediatric Onco-Hematology. Patients with a latency period that was longer than 3 weeks between the last MTX administration of MTX and SLS onset were excluded from the analysis, as were those with unclear etiologies. We assessed symptom severity using a dedicated arbitrary scoring system. Eleven patients were included in the study.

**Results:**

The underlying disease was acute lymphoblastic leukemia type B in 10/11 patients, while fibroblastic osteosarcoma was present in a single subject. The median age at diagnosis was 11 years (range 4–34), and 64% of the patients were women. Symptoms occurred after a mean of 9.45 days (± 0.75) since the last MTX administration and lasted between 1 and 96 h. Clinical features included hemiplegia and/or cranial nerves palsy, paraesthesia, movement or speech disorders, and seizure. All patients underwent neuroimaging studies (CT and/or MRI) and EEG. The scoring system revealed an average of 4.9 points (± 2.3), with a median of 5 points (maximum 20 points). We detected a linear correlation between the severity of the disease and age in male patients.

**Conclusions:**

SLS is a rare, well-characterized complication of MTX administration. Despite the small sample, we have been able to confirm some of the previous findings in literature. We also identified a linear correlation between age and severity of the disease, which could improve the future clinical management.

## Introduction

Methotrexate (MTX) is an antimetabolite agent acting as a competitive inhibitor of the enzyme dihydrofolate reductase (DHFR), hence blocking the synthesis of folate and tetrahydrofolate and inhibiting DNA synthesis during the S-phase of the cell cycle. Folate antagonists were among the first developed antineoplastic agents, and methotrexate is still a mainstay of treatment for leukemia, lymphomas, gastric, breast, and bladder cancer. Neurological complications of anticancer therapy may either result from direct neurotoxicity or from indirect drug-induced metabolic derangements, cerebrovascular disorders or, in the case of checkpoint inhibitors, autoimmune disorders. MTX yields poor drug penetration across the blood–brain barrier due to its ionization and hydrophobicity (1); neurological toxicity mostly results from intrathecal (IT) administration or from high-dose intravenous (IV) treatments leading to intrathecal inflow.MTX neurotoxicity syndrome may cause acute, subacute, or chronic symptoms (2, 3). The overall incidence of MTX neurotoxicity ranges from 3 to 10% and varies according to dose, route, and frequency of administration (4, 5). Factors are high-dose therapy, intrathecal route, young age, and cranial irradiation (6).

While delayed/chronic neurotoxicity may take from months to years to manifest as leukoencephalopathy, acute to subacute neurotoxicity usually occurs within hours to weeks after MTX administration (7). In particular, the weekly or biweekly administration of high-dose MTX (HD-MTX), a prolonged low-dose oral treatment, and IT administration may produce a subacute MTX neurotoxicity called stroke-like syndrome (SLS), possibly characterized hemiplegia, hemisensory deficits, aphasia, dysarthria, dysphagia, diplopia, and occasionally seizures (2). Symptoms develop approximately 2–14 days after drug administration, they last from 15 min to 72 h, and then resolve spontaneously without sequelae. Watanabe et al. observed that the neurological events did not occur immediately after the first IT-MTX administration but started about 1–2 weeks after IT-MTX administration and often fluctuated until they resolved completely (8). Neuroimaging studies are usually normal, although changes have been described on MRI, such as areas of restricted diffusion on diffusion-weighted imaging and non-enhancing T2 hyper-intense lesions in the white matter. Additional investigations such as CSF analysis, and haematologic exams (e.g., thrombophilic profile) are usually normal, whereas electroencephalography (EEG) might show diffuse slowing of the background activity (9, 10). Treatment may consist of observation and supportive care alone. Dextromethorphan, dexamethasone, aminophylline and folic acid have also been successfully employed in MTX neurotoxicity (11–15), such as SLS. However, as most of these cases resolve spontaneously, the value of these medications is not clear. MTX has been eliminated from the therapeutic regimen in most of the cases of subacute neurotoxicity, but cases have also been reported in which further doses of MTX were given without any complication (15).

The incidence of subacute neurotoxicity is still unclear; in 369 children with diagnosed acute lymphoblastic leukemia (B-ALL) treated with both IV-MTX and/or IT-MTX, subacute encephalopathy occurred in 14 patients (3.8%) (16). In other studies, the incidence was higher, ranging from 0.8 to 3.8% (16–18), whereas in a recent paper the incidence of SLS was much lower (0.2%) (19).

## Materials and Methods

We retrospectively recruited 11 patients aged 4–34 years (median 11.02) from the Pediatric Onco-Hematology Centers of Pisa, Bologna, Pavia, and Cagliari who had been diagnosed with leukemia or osteosarcoma and had presented a stroke-like event, defined as the acute or subacute presentation of 1 or more of the following symptoms after MTX administration: hemiplegia, altered consciousness, seizures, hemianopsia, cranial nerve palsy, unilateral sensory disorders or speech disorders. We attributed the neurological event to MTX-induced stroke-like neurotoxicity if the first neurological symptom occurred within 3 weeks after IT or HD MTX administration once other possible causes had been excluded. Patients with neurological symptoms or patients who had presented with evident signs of symptoms on their first visit were excluded, as were those who had clear extracranial problems (i.e. septic shock). Each patient underwent brain imaging using MRI and/or computed tomography (CT). Electronic medical records were reviewed, and data regarding the mode of MTX administration, the temporal relationship to MTX administration, the type, duration and severity of stroke-like symptoms, and the neurological outcome were recorded.

To assess the severity of the disease, we developed an arbitrary ranking scale based on the clinical judgment assigning a score to a total of 13 observed symptoms (Table 1). Each symptom received a score of 0 (no signs/symptoms); 1 (mild symptoms, with a low impact on daily life) or 2 (major symptom, or potentially life-threatening), with a possible maximum sum score of 20.

**Table 1:** Severity score for each symptom observed.

**Symptom**	**Score**
Hyposthenia	1
Hemiplegia	2
Paresthesia	1
Tongue deviation/Protrusion of oral opening	2
Speech disordes (aphasia and/or dysarthria)	2
Impaired consciousness	2
Deafness	1
Corea	2
Tremor	1
Sialorrhea (or drooling)	1
Isolated VII cranial nerve deficit	1
Seizure	2
Hypertension	2

### Statistical Analysis

**Figure 1:**
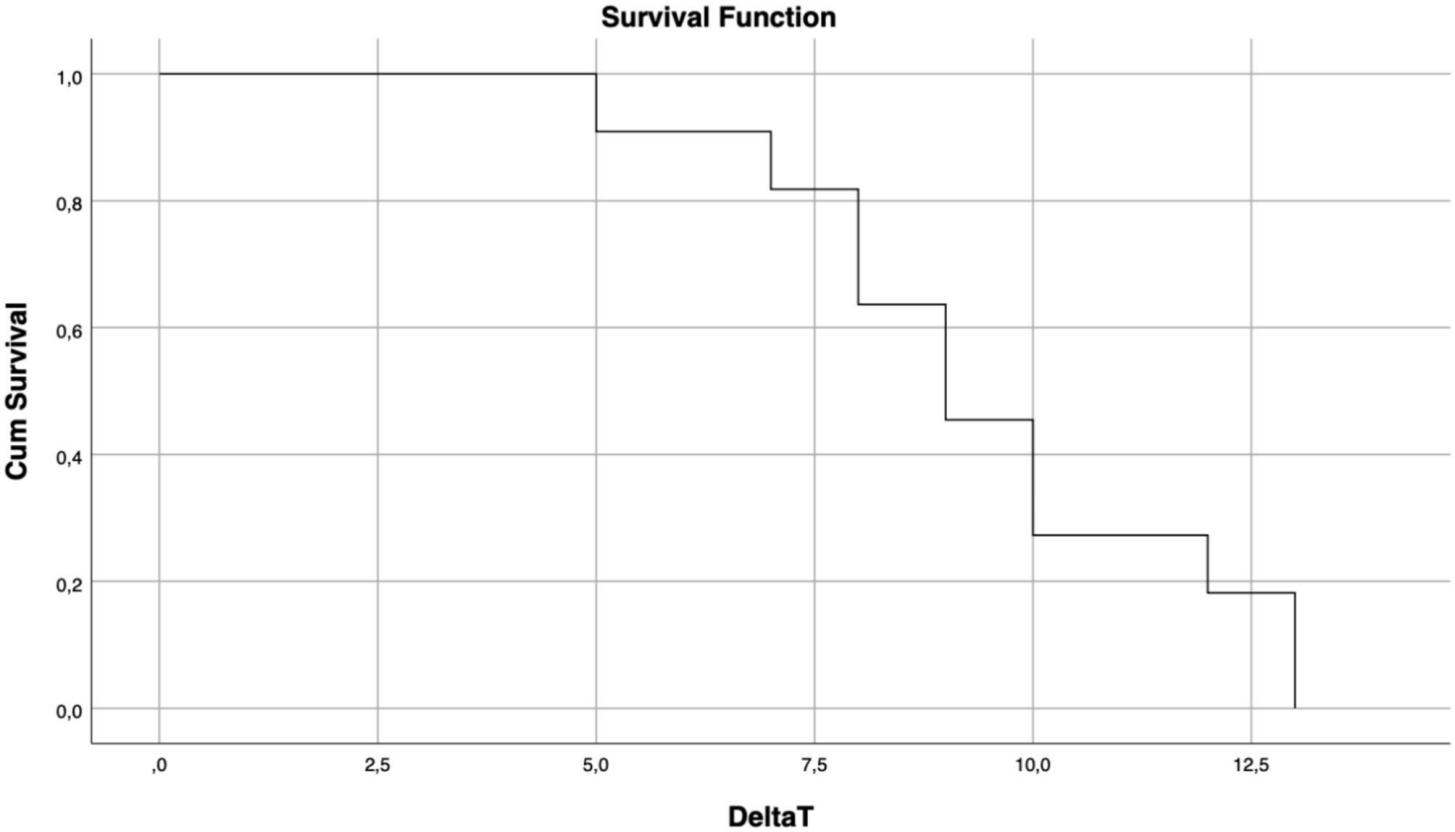
Overall survival rate. Mean follow-up: 68 ± 28 months.

For the statistical analysis of quantitative variables, we evaluated mean, median, and standard deviations, which were compared through *t*-tests. To study the relationship between these variables, the Pearson correlation coefficient was calculated. We described the most relevant correlations with a scatter plot and regression line, calculating the regression coefficient, and the coefficient of determination. The Fisher exact test was employed for the analysis of categorical variables which were expressed as percentages,. Finally, we described the overall survival rates through Kaplan –Meier curves (Figure 1). We used IBM SPSS, ver. 26. for the statistical analysis.

## Results

Total eleven patients with MTX-induced stroke-like neurotoxicity were included in the study. The main characteristics of our cohort are reported in Table 2. Of 11 patients, 64% were women. Of these, ten subjects had acute lymphoblastic leukemia type B (B-ALL) and 1 had fibroblastic osteosarcoma of the right femur. The median age at the time of diagnosis was 11 years (range, 4 – 34 years). None of the patients with SLS showed evidence of CNS leukemia or CNS metastasis at the original presentation. As regards the treatment of patients with B-ALL, 5 (55%) were treated according to the AIEOP-BFM 2009 protocol, and the others (45%) according to the AIEOP-BFM 2017 protocol. The patient with osteosarcoma was treated according to the ISG/OS-2 PGOP NEG protocol. About 6 patients (55%) received IT administration of 12 mg of MTX, 3 (27%) also underwent HD intravenous therapy with 5 g/m (2), only 1 patient received intravenous therapy exclusively, while 1 was treated with oral and IT MTX.

**Table 2:** Clinical data of our study cohort.

**Characteristics**	**Number (%)**
Sex	Male	4 (36%)
	Female	7 (64%)
Age	≤ 10 years old	5 (45%)
	>10 years old	6 (55%)
Diagnosis	LAL-B common	10 (90%)
	Fibroblastic Osteosarcoma	1 (10%)
Involvement of CNS at diagnosis	Yes	0 (0%)
	No	11 (100%)
Trial	AIEOP-BFM 2009	5 (45%)
	AIEOP-BFM 2017	4 (36%)
	ISG/OS-2 PGOP NEG	1 (10%)
	Unknown	1 (10%)

The mean interval between the most recent MTX exposure and SLS was 9.45 days (± 0.75), with a median of 9 days (range 2–13). Figure 1 shows the survival rate of the analyzed population.

SLS episodes occurred in 1 patient during the induction phase, in 4 during re-induction and in 3 patients during the maintenance phase.

Symptoms lasted 24 h in 5 patients, 72 h in 3 patients, and 96 h in 3 patients, as seen in Table 3. The clinical presentation typically included mild paresis and paraesthesias, disturbances of speech and eventually motor impairment

**Table 3:** Main clinical features of our study cohort.

**Patient**	**Sex**	**Tumor**	**Age at onset of SLS**	**Duration (hours)**	**MRI latency (days)**	**MRI findings**	**Therapy adopted**	**Relapse**
1	M	B-ALL	5	72	1	Normal	Heparin, dexamethasone, folinic acid, gabapentin, cefixima	No
2	F	Fibroblastic osteosarcoma	34	6	3	Normal	Amlodipine, nebivolol	No
3	F	B-ALL	6	72	1	Normal	None	No
4	M	B-ALL	7	72	1	White matter hyperintensity in rolandic area	None	No
5	F	B-ALL	13	96	1	Signal alterations in T2 and DWI images in periventricular white matter	None	No
6	M	B-ALL	16	96	3	Hyperintensity in T2-weighted images of deep white matter, DWI showing cytotoxic edema	Midazolam, Trazodone, amlodipine	No
7	F	B-ALL	15	24	2	Hyperintensity in T2-weighted images of deep white matter, DWI showing cytotoxic edema	Midazolam	Yes, after 10 months
8	F	B-ALL	11	96	0	Cytotoxic edema in white matter	Midazolam, folinic acid, Levetiracetam	No
9	F	B-ALL	4	24	0	Hyperintense lesion of the No left corona radiata and bilateral hypeintensity in the frontal white matter in T2-weighted images, without any restriction to diffusion.	Clonazepam	No
10	M	B-ALL	13	24	1	Areas of restricted diffusion with slight hyperintensity in the left centrum semi-ovale and bilaterally in the frontal white matter.	None	No
11	F	B-ALL	9	24	0	Hyperintensity in T2-weighted images of deep white matter, DWI showing cytotoxic edema	None	No

In detail, 7 patients (63.6%) showed limb hyposthenia, 3 (27.3%) had hemiplegia, and 3 (27.3%) developed paraesthesias. In 4 patients (36.4%) tongue deviation and/or protrusion of the buccal opening was observed. About 8 patients (72.7%) had speech disorders, mainly aphasia and/or dysarthria, whereas an altered mental status occurred in 2 cases (18.2%).

Other observed symptoms were deafness, choreic movements, tremors, drooling, or facial nerve palsy. Of these 1 patient experienced seizures and increased blood pressure.

**Figure 2:**
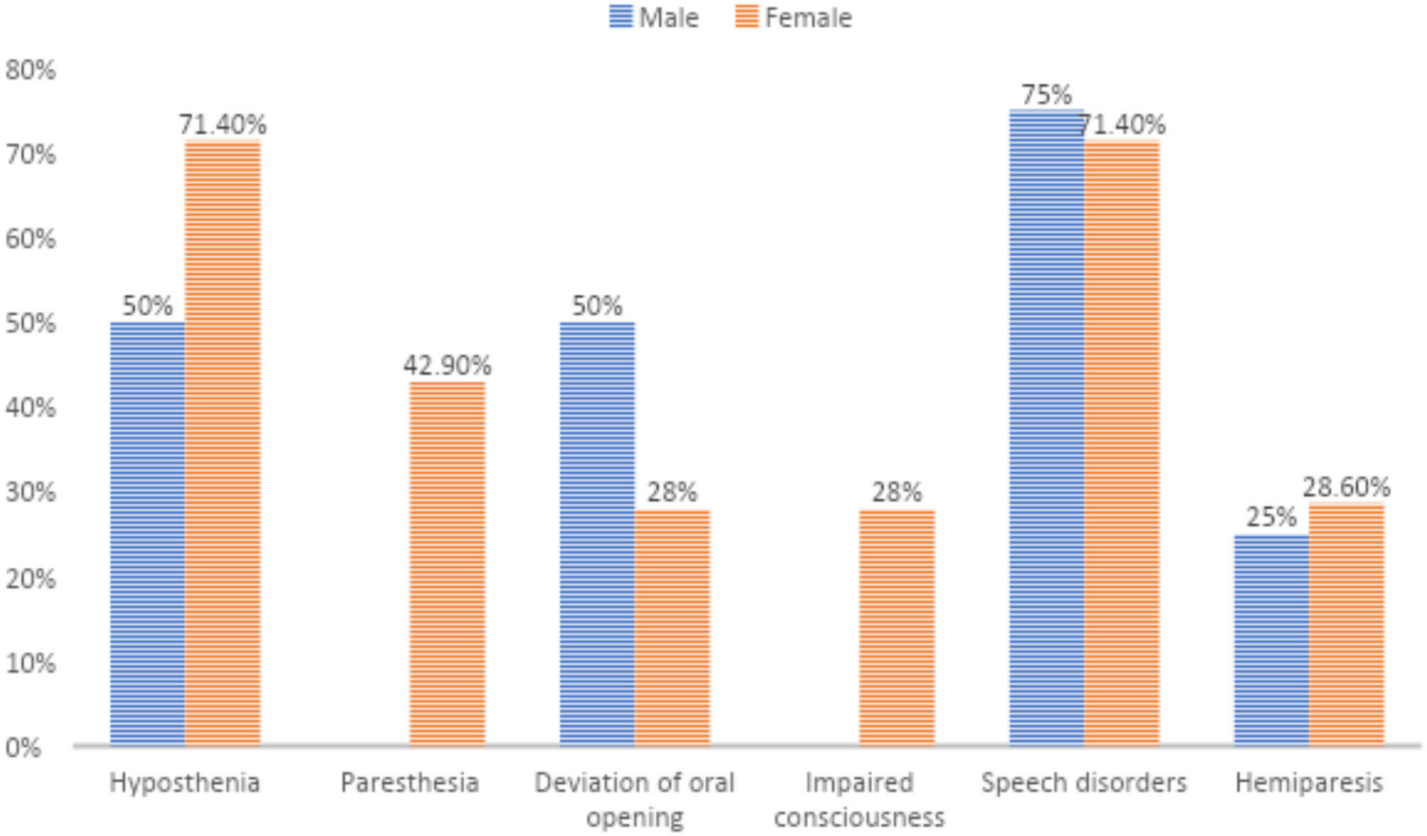
Gender differences in occurrence of symptoms.

The analysis of these symptoms revealed a different gender distribution: limb hyposthenia occurred in 5 (71.4%) women and in 2 (50%) men; no men presented paraesthesia while buccal opening and/or tongue deviation manifested in 28% of females and 50% of males. Impaired consciousness was not observed in men, whereas it occurred in 28% of women (Figure 2).

Our analysis displayed a severity score ranging from 1 to 10, with a mean value of 4.9 (± 2.3), and a median of 5.

**Figure 3:**
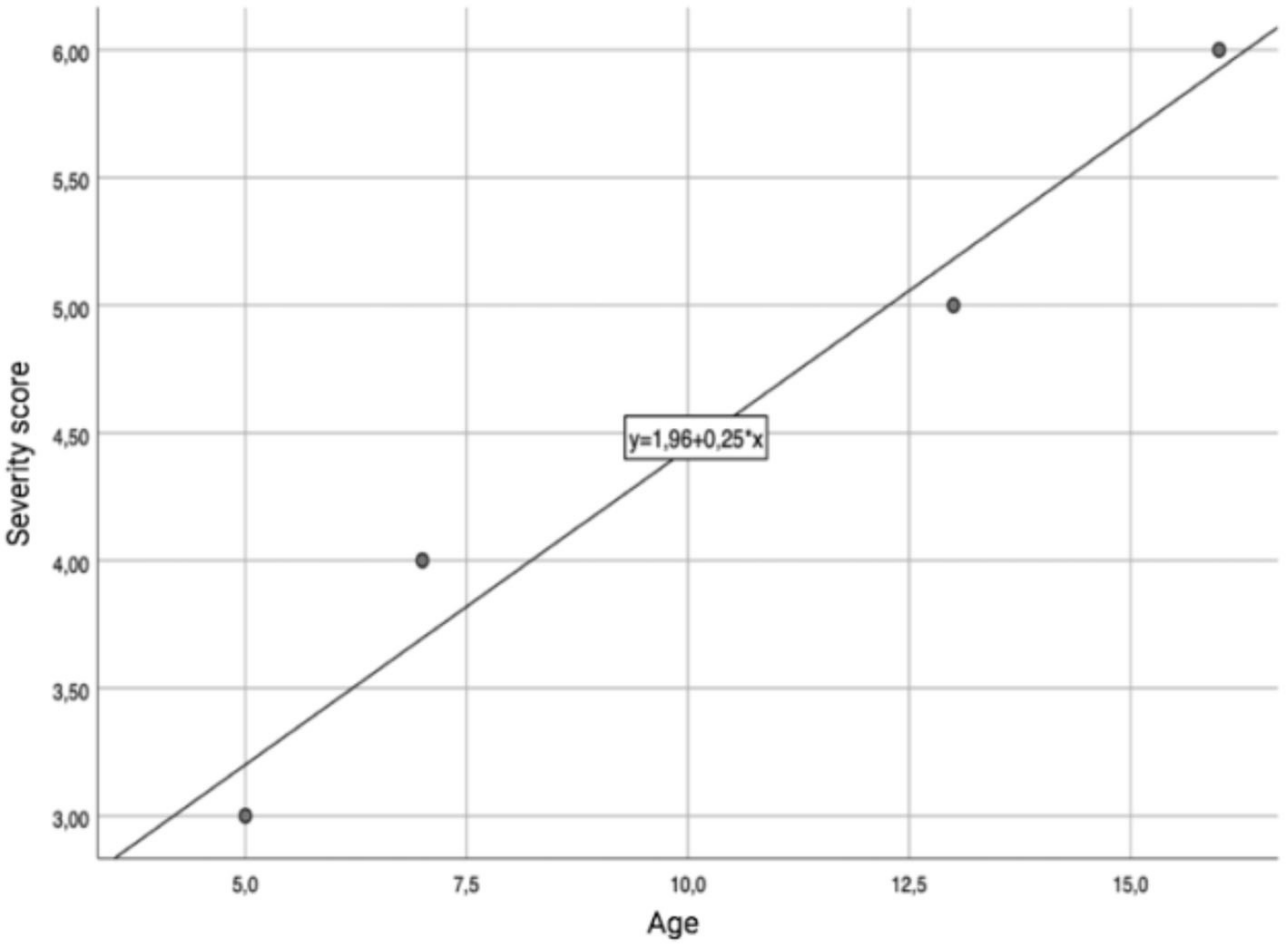
Age-severity linear correlation in males.

**Figure 4:**
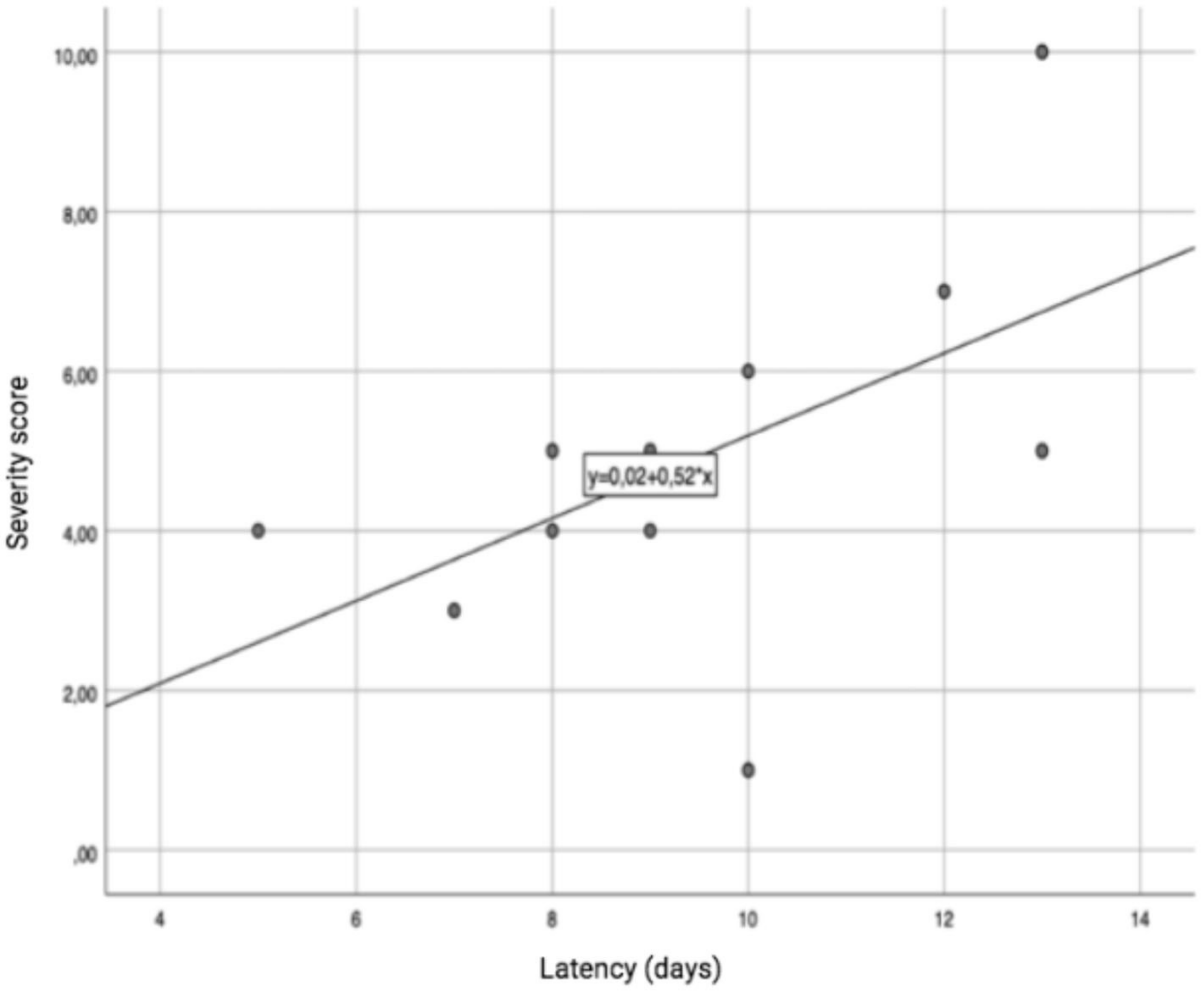
Correlation between the latency time since the last administration of MTX and the severity of the clinical picture.

We did not observe any correlation between the severity of the clinical picture and the age of the female patients, or with the administration modality. On the other hand, a significant correlation was detected in the male subgroup, in which age showed a linear correlation with the severity of the clinical picture (r: 0.98; *p-value*: 0.017), as shown in Figure 3. A trend toward a linear correlation was also observed between severity and the time elapsed since the last administration of MTX (Figure 4), although no statistical significance has been detected (r: 0.56; *p-value*: 0.71).

**Figure 5:**
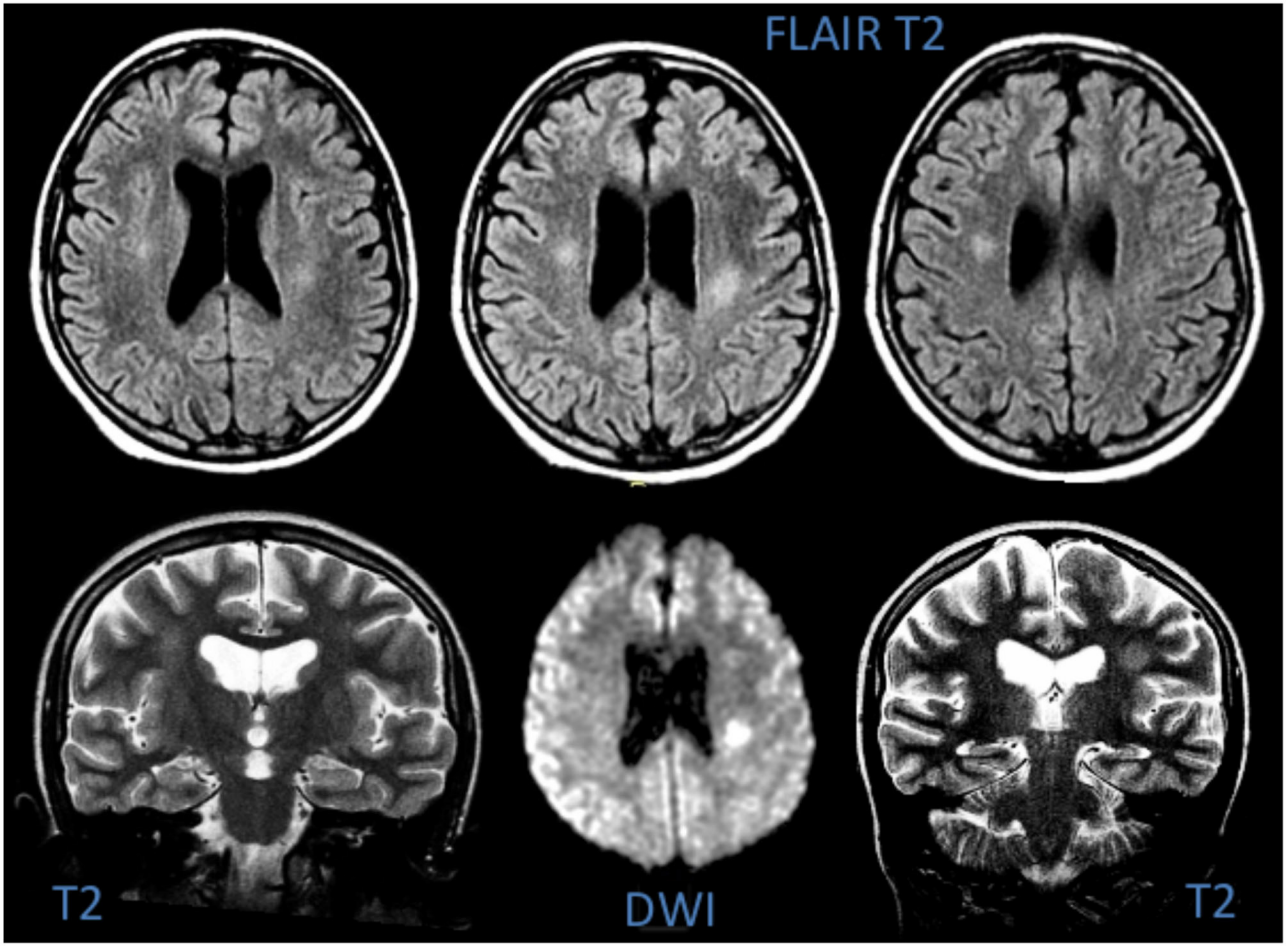
MRI of Patient 6, showing hyperintensity of the deep white matter on T2-weighted images and signs of cytotoxic oedema in DWI.

Total 3 (27%) MRIs were reported as normal, while 8/11 (73%) showed focal or diffuse hyperintensity of the periventricular or subcortical white matter on T2-weighted images; these lesions showed restricted diffusion on ADC map, suggesting cytotoxic oedema (Figure 5). As expected, CT was much less sensitive for white matter changes and was reported as normal in 9/11 cases; only 1/11 patients (Patient 4) presented with mild white matter hypodensity and slight dilation of the liquor spaces which was perhaps due to therapy outcomes. EEG was performed on all patients, and resulted abnormal for mild interhemispheric asymmetry in 2 subjects (Patients 7 and 9). A cerebro-spinal fluid analysis was not performed.

Treatment decisions were made in each case by the local oncology group in joint sessions. As shown in Table 3, 5/11 patients received no therapy, whereas 6/11 patients received pharmacological treatments: 4/11 antiseizure medications (midazolam, clonazepam, levetiracetam), 2/11 (Patients 2 and 6) anti-hypertensive agents (amlodipine, nebivolol), 2/11 (Patients 1 and 8) supplemental folinic acid, *n* = 1/11 (Patient 1), empiric anticoagulant therapy with low-molecular weight heparin, dexamethasone, and gabapentin.

In 5 patients IT-MTX was re-established following the initial SLS. Patients 5 and 6, who experienced clinical symptoms during Protocol M, did not receive the fourth dose of MTX. Patients 4 and 8 discontinued reinduction and phase IB with a subsequent therapy modification. Only 1 patient presented a MTX-induced SLS relapse upon re-exposure during the re-induction therapy (20%), with right paraesthesia, right hemiparesis and dysarthria. The brain MRI showed a T2-weighted hyperintense lesion in the left subcortical white matter. The patient was treated with midazolam with a subsequent remission of symptoms. During maintenance, after 12 doses of oral MTX, the patient re-presented with right hemiparaesthesia and re-flaring of the above mentioned hyperintense lesion. The oncologist decided to stop 6 MP and MTX and to recommence therapy by reducing the dose.

## Discussion

Methotrexate-induced stroke-like syndrome is a rare complication of intrathecal or high dose administration of MTX [≥500 mg/m (2)]. Its peculiar and worrisome clinical features, together with its predisposition to a spontaneous resolution, have elicited the interest of the scientific community since its first descriptions (1). A review of the literature showed 11 reports of neurological serious adverse events associated with MTX administration, determined to be consistent with MTX-induced stroke-like neurotoxicity, characterized by focal neurological dysfunction that could occur with disturbances in speech, vision, or altered mental status, sensorial or motor deficits.

Herein, we examined the main features of such toxicity in a pediatric population through the analysis of a multicentric cohort of 11 patients, so as to highlight the main characteristics of this manifestation and eventually achieve a better understanding of its pathogenesis.

Different hypotheses have been proposed on the mechanisms underlying SLS. Direct damage induced by MTX might result in astrocytosis, axonal loss, and demyelination (20, 21). Several MTX-related biochemical changes could also indirectly affect the central nervous system, such as higher levels of homocysteine and a lower production of methionine resulting from the inhibition of DHFR. Decreased levels of S-adenosylmethionine have been associated with demyelination (22), whereas homocysteine appears to have a direct toxic effect on vascular endothelium (23), elicit oxidative stress (24) and alter coagulation (25), although abnormalities in hemostasis have never been observed in patients with MTX-related neurotoxicity (8, 15).

Moreover, homocysteine-derived metabolites, namely sulfur-containing amino acids, are known to be excitatory agonists of the N-methyl-D-aspartate (NMDA) receptor (26), whose intensive stimulation could lead to seizures and excitotoxicity, eventually leading to neuronal damage and degeneration (27). Furthermore, MTX can decrease the levels of adenosine, biopterins, homovanillic acid, and 5-hydroxyndoleacetic acid (22, 28), which also seem to be involved in the development of neurological alterations (7).

According to the literature, the main risk factors for MTX-induced neurotoxicity include high-dose or intrathecal therapy, association with cranial radiation and age >10 years old (9). Some authors proposed a high MTX/leucovorin ratio as an additional SLS trigger (29).

Our findings support these parameters as risk factors; all of our patients were on IT or HD MTX, and the median age was 11 years (mean: 12 years ± 8.4). However, it is not infrequent to observe SLS in younger patients (8, 30), as we observed in Patient 9. Interestingly, we diagnosed SLS also in a 34-year old patient (Patient 2). The onset of SLS in this patient could be partially explained by the higher doses of MTX used in ISG/OS-2 PGOP NEG protocol and the tendency toward a reduced clearance of MTX in older patients (8). Consistently with this hypothesis, we detected a linear correlation between the age and the severity of the clinical picture in our male patients, which had not been described in previous works. However, no studies have linked SLS with pharmacokinetic parameters of methotrexate.

In our series, the onset of SLS could be observed in induction, re-induction and maintenance treatments. All these phases included the co-administration of Ara-C and Cyclophosphamide. These drugs may promote neurological complications in high-dose regimens, and different authors have hypothesized their role in facilitating MTX-induced neurotoxicity. In particular, we could presume that cyclophosphamide plays a contributing role, since it is not employed in other IT /HD MTX-based schedules which are not characterized by SLS (e.g. trials for acute myeloid leukemia).

Furthermore, SLS was not observed upon the first administration of IT or HD-MTX. This could lead us to presume that there is a possible sensitization or progressive accumulation of this drug as a predisposing factor for its neurotoxicity.

In all our cases, the onset of SLS occurred within 1 or 2 weeks after the last administration of MTX (median 9 days), all patients presented fluctuating symptoms which eventually resolved spontaneously and involved mostly 1 haemisoma. These results were in agreement with those in previous literature. Moreover, 2 patients presented transitory increased blood pressure, 1 diffuse tremor and another patient presented seizures.

The severity of the clinical picture was assessed through an arbitrary scale with a total score ideally ranging from 0 to 20. All patients presented a total score between 1 and 10. According to our observations, the main symptoms presented by our patients determine a significant impairment in their quality of life, such as speech disorders, limb hyposthenia, deviation of the oral opening and hemiplegia.

Interestingly, Patient 6 showed choreic movements. Choreoathetosis had been previously reported in SLS patients and could lead us to presume a possible involvement of basal ganglia.

We failed to identify a correlation between the severity of the clinical picture and the time which had elapsed since the last administration of MTX, although this could be due to the small group of patients.

All patients underwent a CT scan, which was normal in 9/11 cases. Brain MRI, performed between 1 and 3 days after the onset of symptoms, showed a hyperintensity in *centrum semiovale* with restricted diffusion in the same area on T2-weighted sequence. Such findings have been observed in other studies also (8, 31–33), and are similar to those seen in the early phases of cerebral infarction (8). This might suggest that the pathogenesis of SLS could be an ischemic lesion of deep white matter.

Interestingly, 3 of our patients showed a normal MRI. Such findings could be related to inter-subject variability, or to the waxing/waning phase of the disease in which the exam was performed.

MTX administration is associated with higher concentrations of different molecules, even in CSF, such as adenosine and homocysteine, which could lead to damage of the vascular endothelium. It has been therefore hypothesized that the deep white matter, which is less vascularised, may be more prone to developing ischaemic lesions (7).

Moreover, other authors have suggested that the peculiar fluctuating manifestations of SLS might be the sign of a progressive depolarization of neuronal and axonal membranes (1, 9), similarly to migraine-associated cortical spreading depression (CSD). In this case, a pivotal role could be played by the MTX-associated astrocytosis (20, 25). It has been observed that astrocytes may show intracellular calcium waves that spread over long distances (34) and can modulate neuronal and vascular activity.

As regards the treatment of SLS, different drugs could reverse the biochemical effects of MTX (7). Two of our patients received additional folic acid, which may have an effect on the above-mentioned ratio with MTX concentrations, preventing relapses or fluctuations and possibly improving recovery. In both cases, the treatment was followed by remission of the symptoms. Notably, rescue with leucovorin has been successfully employed by some authors in patients rechallenged with HD or intrathecal MTX to prevent relapses of SLS (16). Aminophylline, due to its action as a competitive antagonist of adenosine, has also been widely administered as secondary prophylaxis in patients with MTX-induced stroke-like syndrome (9, 14, 16, 30–32). Moreover, other authors (31, 35), have reported the use of dextromethorphan in some patients. The efficacy of this treatment could be related to its antagonizing action on the NMDA receptor, which might inhibit the excitatory effects of homocysteine metabolites. Total 2 patients required anti-hypertensive therapy. Of these, 1 patient, (Patient. 6), who also presented choreic movements, required the administration of midazolam, the latter being also effectively employed in our patients with tremor and seizures. To our knowledge, no specific treatment has been proposed for SLS besides supporting therapy. Given the predisposition to a spontaneous resolution of symptoms, a gold standard of management is still to be defined.

## Conclusions

Although SLS is a well-known complication of MTX administration, its clinical features and correct treatment are still largely debated. The worrisome clinical picture, which occurs in other complications of chemotherapy, such as Posterior Reversible Encephalopathy Syndrome (36), represents a major concern for the clinician. Better comprehension of the syndrome is therefore needed. Through the analysis of 1 of the largest cohorts of patients in literature, we have been able to achieve a better understanding of the clinical features and severity of MTX-induced SLS. Our results, despite the limited sample, have confirmed some of the main assessments observed in literature thus far. Nonetheless, we have also detected a linear correlation between age and severity of the disease, which could lead to changes in the management of SLS.

## Data Availability Statement

The original contributions presented in the study are included in the article/supplementary material, further inquiries can be directed to the corresponding author.

## Ethics Statement

Ethical review and approval was not required for the study on human participants in accordance with the local legislation and institutional requirements. Written informed consent to participate in this study was provided by the participants' legal guardian/next of kin.

## Author Contributions

AS, AO, GN, IT, and VF contributed to conception and design of the study. AS, GN, IT, and VF organized the database. GM performed the statistical analysis. AS wrote the first draft of the manuscript. AS, GN, IT, EB, AO, and RB wrote sections of the manuscript. All authors contributed to manuscript revision, read, and approved the submitted version.

## Funding

This work has been partially supported by grant-RC 1.22 and the 5 x 1,000 voluntary contributions, Italian Ministry of Health (RB and EB).

## Conflict of Interest

The authors declare that the research was conducted in the absence of any commercial or financial relationships that could be construed as a potential conflict of interest.

## Publisher's Note

All claims expressed in this article are solely those of the authors and do not necessarily represent those of their affiliated organizations, or those of the publisher, the editors and the reviewers. Any product that may be evaluated in this article, or claim that may be made by its manufacturer, is not guaranteed or endorsed by the publisher.
